# Erythropoietin improves motor and cognitive deficit, axonal pathology, and neuroinflammation in a combined model of diffuse traumatic brain injury and hypoxia, in association with upregulation of the erythropoietin receptor

**DOI:** 10.1186/1742-2094-10-156

**Published:** 2013-12-18

**Authors:** Sarah C Hellewell, Edwin B Yan, Dasuni S Alwis, Nicole Bye, M Cristina Morganti-Kossmann

**Affiliations:** 1 National Trauma Research Institute, Alfred Hospital, Level 4, Burnet Tower, 89 Commercial Road, Melbourne, VIC 3000, Australia; 2 Department of Surgery, Monash University, Level 4, Burnet Tower, 89 Commercial Road, Melbourne, VIC 3000, Australia; 3 Department of Physiology, Monash University, Clayton, VIC 3800, Australia; 4 Department of Epidemiology and Preventive Medicine, Monash University, Level 5, Burnet Tower, 89 Commercial Road, Melbourne, VIC 3000, Australia; 5 Australian New Zealand Intensive Care Research Centre, Level 5, Burnet Tower, 89 Commercial Road, Melbourne, VIC 3000, Australia; 6 Barrow Neurological Institute, Department of Child Health, University of Arizona, Level 5, Burnet Tower, 89 Commercial Road, Melbourne, VIC 3000, Australia

**Keywords:** Traumatic brain injury (TBI), Traumatic axonal injury, Hypoxia, Erythropoietin, EPO, Neuroprotection

## Abstract

**Background:**

Diffuse axonal injury is a common consequence of traumatic brain injury (TBI) and often co-occurs with hypoxia, resulting in poor neurological outcome for which there is no current therapy. Here, we investigate the ability of the multifunctional compound erythropoietin (EPO) to provide neuroprotection when administered to rats after diffuse TBI alone or with post-traumatic hypoxia.

**Methods:**

Sprague–Dawley rats were subjected to diffuse traumatic axonal injury (TAI) followed by 30 minutes of hypoxic (Hx, 12% O_2_) or normoxic ventilation, and were administered recombinant human EPO-α (5000 IU/kg) or saline at 1 and 24 hours post-injury. The parameters examined included: 1) behavioural and cognitive deficit using the Rotarod, open field and novel object recognition tests; 2) axonal pathology (NF-200); 3) callosal degradation (hematoxylin and eosin stain); 3) dendritic loss (MAP2); 4) expression and localisation of the EPO receptor (EpoR); 5) activation/infiltration of microglia/macrophages (CD68) and production of IL-1β.

**Results:**

EPO significantly improved sensorimotor and cognitive recovery when administered to TAI rats with hypoxia (TAI + Hx). A single dose of EPO at 1 hour reduced axonal damage in the white matter of TAI + Hx rats at 1 day by 60% compared to vehicle. MAP2 was decreased in the lateral septal nucleus of TAI + Hx rats; however, EPO prevented this loss, and maintained MAP2 density over time. EPO administration elicited an early enhanced expression of EpoR 1 day after TAI + Hx compared with a 7-day peak in vehicle controls. Furthermore, EPO reduced IL-1β to sham levels 2 hours after TAI + Hx, concomitant to a decrease in CD68 positive cells at 7 and 14 days.

**Conclusions:**

When administered EPO, TAI + Hx rats had improved behavioural and cognitive performance, attenuated white matter damage, resolution of neuronal damage spanning from the axon to the dendrite, and suppressed neuroinflammation, alongside enhanced expression of EpoR. These data provide compelling evidence of EPO’s neuroprotective capability. Few benefits were observed when EPO was administered to TAI rats without hypoxia, indicating that EPO’s neuroprotective capacity is bolstered under hypoxic conditions, which may be an important consideration when EPO is employed for neuroprotection in the clinic.

## Background

Diffuse axonal injury (DAI) is increasingly recognised as a prominent cause of morbidity and mortality after traumatic brain injury (TBI) [1]. DAI is often complicated by the co-occurrence of secondary hypoxia [2,3], arising from brain stem injury, airway obstruction, acute respiratory distress, or cerebral hypoperfusion/hypoventilation [4,5]. Epidemiological studies describe post-traumatic hypoxia as common sequelae to diffuse injury, worsening neurological outcome [3,6,7]. Experimentally, hypoxia has been found to aggravate behavioural outcomes [8-10], exacerbate oedema and blood brain barrier (BBB) dysfunction [11-13], heighten axonal pathology [14,15], and prolong cerebral inflammation [10,15].

Despite earnest research, no effective therapy has been developed for the treatment of TBI, with a multitude of compounds that showed promise in animal studies failing to exhibit beneficial effects in clinical trials. To date, more than 20 compounds have been assessed in phase II/III trials, with none showing long-term benefit [16]. This lack of success in translation from the laboratory to the clinic has been attributed to several factors, including variations in therapeutic doses and administration windows in animals versus humans, premature passage to the clinic, failure of experimental models to include secondary insults that are commonplace in clinical TBI, and importantly, a lack of research into specifically axonoprotective molecules in diffuse TBI models [17].

The haematopoietic agent erythropoietin (EPO) has garnered interest in recent years as a potential neuroprotective treatment in various neuropathologies, in which EPO has been found to confer a wide range of benefits on a functional, cellular and biochemical level [18]. Although Epo and its cognate receptor EpoR are endogenously expressed and upregulated under injurious conditions such as ischemia, hypoxia or TBI [19-21], their levels of expression rarely appear sufficient to curtail tissue damage and promote neurological recovery [22]. Therefore, pharmacological treatment with exogenous EPO after CNS injury has been a keen area of investigation, particularly with respect to focal brain injury, where EPO has shown to be efficacious in improving sensorimotor and spatial memory after both controlled cortical impact injury (CCI) and cryogenic lesion [22-25], as well as minimising BBB dysfunction and oedema [23,26]. EPO is also a potent immune modulator, and is able to reduce levels of the pro-inflammatory cytokines NFκB, IL-1β, TNF-α, ICAM-1 and CCL-2 in comparison to vehicle-treated rats when assessed post cortical contusion injury or lateral fluid percussion injury [26-29].

While there is now ample evidence that EPO is beneficial following focal CNS injury, little is known as to the effects of EPO in limiting the pathology of diffuse TBI, the most complex and relevant form of brain trauma, which is associated with high rates of morbidity and lifelong devastating impairments. To date, the therapeutic effects of EPO have been examined predominantly in an acute context after diffuse TBI using carbamylated EPO (CEPO), with administration 30 minutes after impact acceleration injury shown to decrease nitric oxide synthesis at 1 hour, with a subsequent attenuation of oedema as detected by diffusion-weighted MRI from 2 hours [30-32]. This one early dose of CEPO was also sufficient to improve neurological function when examined out to 10 days postinjury [31].

Due to the low risk of adverse outcome and high potential for benefit, EPO is currently under phase III clinical trial for TBI with results eagerly anticipated. However, to date, no studies have examined functional or cellular changes in experimental diffuse TBI treated with EPO beyond the acute setting, and crucially, no studies have explored the potential for EPO to benefit when TAI is combined with secondary hypoxia. In light of these findings, we investigated the ability of EPO to improve outcome after experimental diffuse TBI both alone and in the context of post-traumatic hypoxia in order to elucidate EPO’s effects on behavioural and cognitive function, axonal and dendritic integrity, inflammation, and changes in the expression of endogenous Epo receptor.

## Methods

### Model of diffuse traumatic brain injury, post-traumatic hypoxia and erythropoietin therapy

Animal experiments were conducted in accordance with the Code of Practice for the Care and Use of Animals for Scientific Purposes (National Health and Medical Research Council, Australia), and received approval from the Alfred Medical Research and Education Precinct (AMREP) Animal Ethics Committee. Adult male Sprague–Dawley rats were housed under a 12-hour light/dark cycle with food and water *ad libitum*.

The Marmarou model of diffuse traumatic axonal injury (TAI) was used in this study as described by others and our group [10,15,33]. Rats aged 12 to 16 weeks and weighing 350 to 375 g on the day of surgery were randomly allocated to one of eight treatment groups, including TAI (n = 30), TAI followed by a 30-min systemic hypoxia (TAI + Hx; n = 30), hypoxia only (n = 30) or sham surgery (n = 30). After surgery, an equal number of rats from each group were divided into either vehicle or EPO treatment. As described previously [15], rats were anaesthetised with a mixture of 5% isoflurane in 22% O_2_/78% N_2_, endotracheally intubated and mechanically ventilated with a maintenance dose of 2 to 3% isoflurane in 22% O_2_/78% N_2_. A steel disc of 10-mm diameter and 3-mm thickness was adhered to the skull between the bregma and lambda suture lines using dental acrylic. Rats were briefly disconnected from the ventilator and placed on a foam mattress (Type E polyurethane foam, Foam2Size, Ashland, VA, USA) underneath the trauma device, and a weight of 450 g was allowed to fall freely through the vertical tube from a height of 2 m. Following the impact, rats were reconnected to the ventilator for a further 30 min using an appropriate concentration of isoflurane (0.5 to 1%) in either hypoxic (12% O_2_/88% N_2_) or normoxic (22% O_2_/78% N_2_) conditions. We have previously reported the consistent physiological consequences of this insult resulting in a sO_2_ of 47 ± 4.3% for the duration of hypoxia, with a concomitant episode of hypotension, whereby mean arterial blood pressure was reduced to 69.5 ± 29.5 mmHg [15]. Hypoxia-only and sham-operated animals were surgically prepared as described for TAI rats with the exception of the traumatic impact, and ventilated with hypoxic or normoxic gas, respectively. After surgery, rats were housed in individual cages and placed on 37°C heat pads for 24 h to maintain normothermia during recovery.

### Administration of erythropoietin

At 1 and 24 hours after injury rats were injected intraperitoneally (i.p.) with either recombinant human (rh)EPO-α (5000 IU/kg; Janssen-Cilag, Australia) (henceforth referred to as EPO) or the equivalent dose of saline as previously described [22,27,32]. This dual acute/subacute dosing schedule was chosen due to the respective benefits of resolution of tissue pathology observed with acute administration of EPO [34], and the behavioural benefits observed with a subacute dose [35]. Rats designated for a 2- or 24-hour survival time received EPO or saline i.p. 1 hour postinjury only.

### Assessment of sensorimotor and cognitive function

#### Rotarod

Six rats from each treatment group at each time point were assessed for sensorimotor deficit using the Rotarod test [36], in which rats must climb on a rotating cylinder comprising 18 rods (1 mm diameter) (Ratek, Boronia, VIC, Australia), with the rotational speed increasing in increments of 3 revolutions per minute (rpm) per 5 sec, from 0 to 30 rpm. The maximal speed recorded when the rat was unable to match and slipped from the Rotarod was taken as the score for each trial. On the week before surgery, rats were trained on the Rotarod every second day. After injury, rats were assessed daily for one week, and every second day for the second week, concluding at 14 days.

#### Open field exploration

The open field test was used to assess locomotor activity as previously described [10]. A separate group of rats were generated specifically for this and the novel object recognition test (n = 4/group) and were not used for assessment on the Rotarod or for subsequent histological analysis. At 6 days postinjury, rats were placed in an empty arena (70 × 70 × 60 cm, W × L × H) within a dimly lit, closed environment. Rats were allowed to explore freely for a period of 15 minutes, with their movement tracked by camera and the distance walked calculated using a custom-made automated movement-tracking program.

#### Novel object recognition test

The novel object recognition test (NORT) examines short-term episodic recognition memory [37], relying on the implicit exploratory activity of rodents to discern between a novel object and one previously encountered [37-39]. Rats were assessed on day 6 postsurgery, and first habituated to the arena for 15 minutes. Upon conclusion, rats were removed briefly (less than 1 min), while two identical objects were placed 10 cm away from the walls in opposing corners of the arena. The rats were placed back into the experimental chamber and allowed to explore the objects freely for 15 min (learning phase). After this, rats were returned to their home cages for a retention delay of 1 h, during which one familiar object was replaced with a novel one. Subsequently, rats were again placed in the area and allowed to explore the objects for 15 min. Object exploration was scored as nose or forepaw contact within an area of 2 cm encircling each object. Rats exploring for less than a cumulative total of 15 seconds were omitted from analysis (n = 2). Between trials, objects were cleaned thoroughly with 70% ethanol to eliminate olfactory cues.

### Tissue collection

At 1, 7 and 14 days after treatment, those rats previously assessed on the Rotarod (n = 6 per group per time point) were killed, and brains were perfusion-fixed using 4% paraformaldehyde and embedded in paraffin wax. Brain tissue blocks were cut into 10-μm thick coronal sections at the level of +0.2 mm and -11.6 mm relative to bregma and collected onto glass slides.

A separate cohort of rats was generated for assessment of IL-1β concentration at 2, 24 and 48 h postinjury in brain homogenates (n = 4 per group per time point). Brains were divided into left and right hemispheres, the cortex and cerebellum removed, and the remaining tissue comprising brainstem, corpus callosum, hippocampus, thalamus and hypothalamus was combined to from a crude white matter preparation, and stored at -80°C until use. This allowed for measurement of IL-1β in brain regions more susceptible to diffuse injury and axonal damage.

### Measurement of the corpus callosum area

Three consecutive brain sections were dewaxed, rehydrated, stained using hemotoxylin and eosin, and visualised under a light microscope (Olympus BX50; Olympus, Mt Waverley, VIC, Australia). Brains were photographed at low power (2x objective) to cover the entire section. The whole brain area and the area of the corpus callosum were measured using ImageJ (v. 1.45; National Institutes of Health, Bethesda, MD, USA), and the area of the corpus callosum was expressed as the percentage of total brain area.

### Immunohistochemistry

Immunohistochemistry was performed on three consecutive sections per region of interest per animal using the following primary antibodies: neurofilament heavy-chain (NF-200, 1:1000, Zymed, Carlsbad, CA, USA), microtubule-associated protein 2 (MAP2, 1:200, Millipore, North Ryde, N.S.W., Australia), membrane-bound EPO receptor (EpoR, 1:150, Santa Cruz Biotechnology, Dallas, TX, USA), CD68 (1:150, Millipore). All staining was visualised using diaminobenzidine (DAB) as the chromagen, following incubation with appropriate secondary antibodies and reagents provided in the peroxidase ABC kit (Vector Laboratories, Burlingame, CA, USA) as described earlier by our group [15].

To determine which cell types expressed EpoR and whether EPO treatment induced EpoR expression, double labelling immunofluorescence was performed using antibodies to EpoR in conjunction with NF-200, CD68, NeuN (neurons, 1:1000, Chemicon, Billerica, MA, USA) or GFAP (astrocytes, 1:1000, Dako Cytomation, Glostrup, Denmark). Sections were incubated simultaneously with each marker overnight at 4°C, and labelled cells were visualised by incubation with fluorescent-tagged secondary antibodies (1:200 goat anti-rabbit Alexa 594 (red) and 1:200 goat anti-rabbit Alexa 488 (green); Invitrogen, Carlsbad, CA, USA).

### Analysis of immunohistochemistry

All sections stained with NF-200, MAP2, EpoR and CD68 were visualised using light microscopy, with the exception of double labelling experiments, which were analysed using fluorescence microscopy (Olympus BX50).

Brain sections were photographed using a digital camera (Olympus DP71) with AnalySIS life science software (Olympus). Quantitative analysis of the micrographs was performed using ImageJ software. Multiple photographs were taken to cover the entire corpus callosum (for NF-200, EpoR and CD68) and lateral septal nucleus (MAP2) at the level of +0.2 mm to bregma, and pyramidal tracts at -11.6 mm to bregma) in the case of NF-200 and EpoR. Three sections were examined per region for each brain, with cell counts (in the case of NF-200, EpoR and CD68) averaged per section for each animal. Densitometric analysis of MAP2 immunohistochemistry was performed in a region of interest in the lateral septal nucleus, corresponding to an area of 1.43 mm^2^. Measurements were performed over three consecutive sections per brain using ImageJ software.

### IL-1β measurement

White matter preparations were homogenised in an extraction solution containing Tris–HCl (50 mmol/L, pH 7.2), NaCl (150 mmol/L), 1% Triton X-100, and 1 μg/mL protease inhibitor cocktail (Complete tablet; Roche Diagnostics, Basel, Switzerland) and agitated for 90 min at 4°C. Tissue homogenates were then centrifuged at 2,000 rpm for 10 min, and the supernatant stored at -80°C until use. The concentration of IL-1β was determined using a rat IL-1β ELISA (R&D Systems, Minneapolis, MN, USA) with samples run undiluted in duplicate, and incubated on pre-coated plates. The optical density of each well was measured at 450 nm, with a wavelength correction of 540 nm (as suggested by the manufacturer). The concentration of IL-1β was calculated against the standard curve. Total protein concentrations were determined for each sample using the Bradford Assay (Bio-Rad Laboratories, Hercules, CA, USA) and the concentration of IL-1β expressed as picogram per milligram of total protein.

### Data analysis

Statistical analysis was performed using a commercially available software package (GraphPad Prism version 5.0 for the Macintosh; GraphPad, La Jolla, CA, USA). Results from the Rotarod test were analysed using a two-way repeated measures ANOVA, and where initial significant changes were observed with regards to treatment, time and interaction between the two factors, Bonferroni post-hoc analysis was performed. Immunohistochemistry, callosal area and IL-1β data were analysed by two-way ANOVA, also with Bonferroni post-hoc analysis where appropriate. Data from the open field exploration was analysed by one-way ANOVA with post-hoc Bonferroni analysis, while preferences for familiar versus novel objects in the NORT was analysed using a *t*-test. Data were presented as mean ± standard error of the mean and were considered significant where *P <0.05. P* values are indicative of Bonferroni post-hoc analysis unless otherwise indicated.

## Results

### Erythropoietin promotes significant improvement in motor function after TAI + Hx

**Figure 1:**
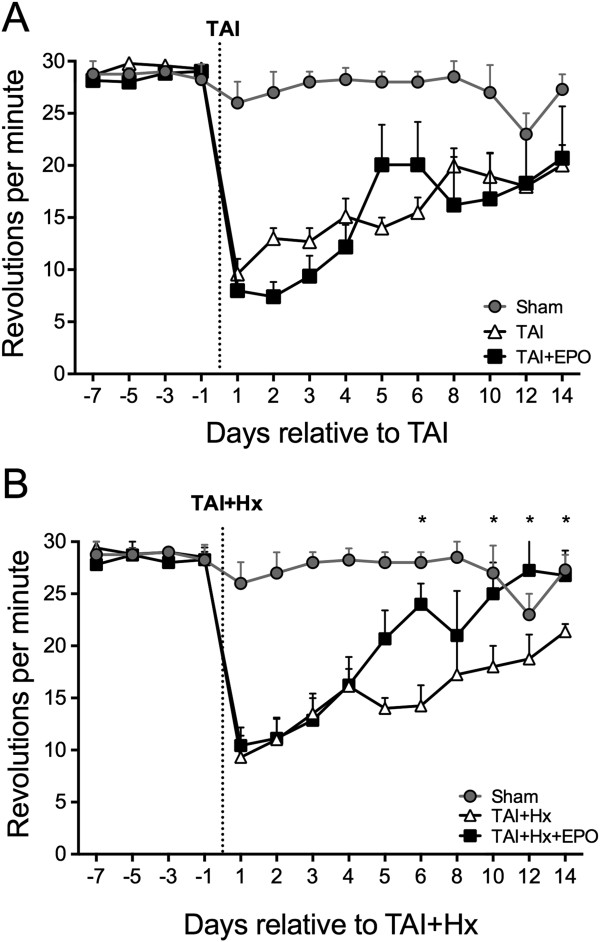
**Erythropoietin (EPO) promotes significant improvement in motor function in rats subjected to traumatic axonal injury with hypoxia (TAI + Hx), but not in traumatic axonal injury (TAI) alone.** Rats were trained on the Rotarod task for 7 d prior to injury, then tested daily for 6 d after surgery and on every second day until 14 d. **(A)** The deficit observed after TAI and TAI + EPO rats was similar 1 d postinjury. TAI rats made a steady recovery and improved throughout the course of the 2-week testing period; however there was no additional improvement after EPO treatment. **(B)** Both TAI + Hx and TAI + Hx + EPO groups achieved identical rpm scores on the Rotarod until 4 d postinjury, from which point TAI + Hx + EPO rats were performing significantly better on the Rotarod by 6 d than TAI + Hx rats. By 10 d, TAI + Hx + EPO rats reached an rpm similar to sham, with this beneficial effect maintained out to the end of the testing period. Data shown as mean ± SEM. N at 1 day = 24/group; 2 to 6 days = 12/group; 8 to 14 days = 6/group. Data was analysed by 2-way ANOVA repeated measures with Bonferroni post hoc test, with a *P* value of <0.05 considered significant.

Both TAI and TAI + Hx treatment groups demonstrated a severe sensorimotor deficit on the Rotarod 1 day postinjury when compared to sham, with scores of 9.58 ± 1.40 rpm and 9.3 ± 2.09 rpm (Figure 1A,B, respectively). Similar scores were also achieved at this time when either group was administered EPO. When Rotarod scores were assessed across groups using two-way RM ANOVA, there was a significant effect of treatment and time, and a significant interaction between the two (*P <0.001*).

As shown in Figure 1A, over the course of 14 days TAI rats steadily improved in motor function. TAI + EPO rats showed a comparable degree of recovery in their Rotarod performance compared to their TAI-vehicle counterparts, with scores remaining similar in each group until 14 days. The Rotarod scores observed in TAI + Hx and TAI + Hx + EPO rats were also similar to each other and to the TAI groups at 1 day (9.3 ± 2.09 rpm versus 10.44 ± 1.73 rpm, respectively, Figure 1B), with an equal rate of improvement between groups until day 4. During this acute phase, the maximal speed achieved by each TAI + Hx group was significantly slower than sham control rats (day 4: 16.12 ± 1.66 rpm, TAI + Hx and 16.23 ± 2.7 rpm, TAI + Hx + EPO versus 28.25 ± 1.12 rpm, sham; *P <0.05).* However, at 5 days postinjury TAI + Hx + EPO rats made a remarkable functional improvement, and at 6 days were performing significantly better on the Rotarod than the TAI + Hx rats (24.0 ± 2.0 versus 14.25 ± 1.98, *P <0.05*). By 10 days, TAI + Hx + EPO rats had returned to sham-level scores (*P <0.05*), with significant differences to TAI + Hx rats on days 10, 12 and 14. Conversely, untreated TAI + Hx rats showed a plateau in recovery at 8 days post-injury, with scores of 17.25 ± 3.94 rpm, and still maintained a significantly greater deficit compared to sham (28.5 ± 1.50 rpm, *P <0.05*).

### Erythropoietin improves travel distance in open field exploration in TAI + Hx rats

**Figure 2:**
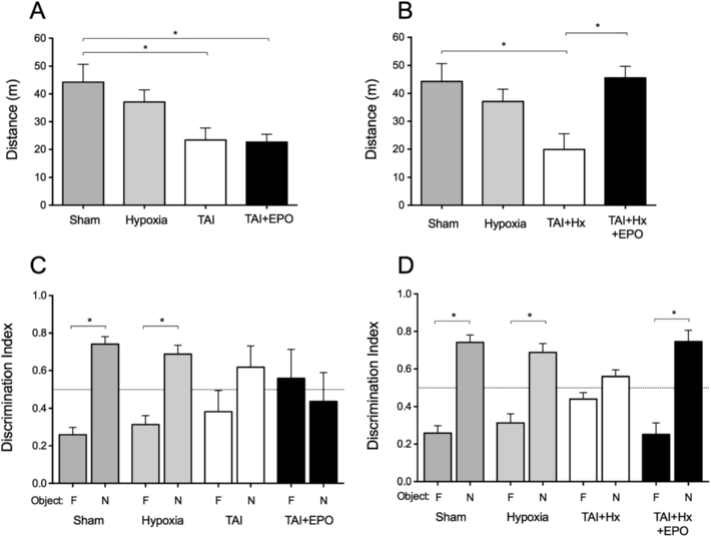
**Erythropoietin (EPO) therapy improves motor deficit and restores spatial memory in rats with post-traumatic axonal injury (TAI) hypoxia (Hx). (A)** At 6 d postinjury, TAI rats travelled significantly shorter distances in the testing arena over 15 min with or without EPO treatment. **(B)** TAI + Hx rats covered slightly shorter distances than TAI rats, and were significantly impaired compared to sham. However, when treated with EPO, TAI + Hx rats had a remarkable recovery, with distances traveled significantly improved to those seen in sham rats. **(C)** The novel object recognition test (NORT) was used to assess memory retention. **(A)** In the recall phase of testing, sham and hypoxia treated rats spent a significant proportion of their total exploration time on the novel object however this recognition was lost after TAI. Administration of EPO to TAI rats did not improve the proportion of time spent exploring the novel object. **(D)** TAI + Hx rats also appeared to have no recollection of the familiar object, spending identical proportions of time exploring both the novel and familiar objects over 15 min. EPO significantly reversed memory dysfunction and returned values back to those observed for sham. Data are expressed as mean ± SEM, n = 4/group. *P <0.05 when analysed by 1-way ANOVA with Bonferroni post-hoc test.

When assessed in the open field at 6 days postsurgery, sham and hypoxia rats travelled a similar distance within 15 min (Figure 2A). While after TAI, rats showed a significant impairment, and were only able to travel half of the distance achieved by sham animals (23.38 ± 4.36 m versus 44.26 ± 6.40, respectively, one-way ANOVA: effect of treatment *P = 0.0068*; Bonferroni post hoc, *P <0.05*). EPO treatment failed to improve this deficit in TAI + EPO rats, which maintained distances travelled identical to TAI vehicle control animals (*P <0.05*).

The addition of hypoxia after TAI further reduced the distance travelled below that of TAI alone by 15%, with a mean distance of 19.90 ± 5.62 m; a value also significantly below that observed for sham rats (*P <0.05*, Figure 2B). Conversely, when treated with EPO, TAI + Hx rats had a significant increase in their travel distances to more than double that of their vehicle- treated counterparts (*P <0.05*), which returned them to sham values.

### Erythropoietin improves recognition of the novel object after TAI + Hx, but not after TAI alone

In the learning phase, rats of all groups showed no preference for either of the identical objects with all injured rats exploring for approximately half of the total exploration time of sham controls whether treated with vehicle or EPO (data not shown). As depicted in Figure 2C, in the recall phase of testing, sham rats spent a significantly larger proportion of time exploring the novel object compared to the familiar, with a mean value of 74% of total exploration spent on the novel object, and only 24% spent on the familiar object (*P <0.001*, familiar (F) versus novel (N), one-way ANOVA with Bonferroni post hoc analysis). Rats exposed to hypoxia alone also spent a significant proportion of time exploring the novel object compared to the familiar, with near-identical ratios to those observed for sham (*P <0.05*, F versus N). Conversely, TAI rats spent a similar proportion of time exploring the novel object and familiar, with no significant difference observed between TAI rats with (*P = 0.72, F versus N*) or without EPO treatment (*P = 0.27, F versus N*, Figure 2C).

Similarly, to TAI, rats subjected to TAI + Hx did not show an object preference and spent similar proportions of time exploring both the familiar and the novel object (*P = 0.76,* F versus N, Figure 2D), presenting scores of 45% and 55% for the familiar and novel objects, respectively. However, when TAI + Hx rats were administered EPO, a substantial shift in memory function was observed, with rats spending a significantly elevated proportion of time exploring the novel object for 75% of the total exploration time when compared to the previously encountered object (*P <0.05*, F versus N).

### TAI and TAI + Hx induced atrophy of the corpus callosum is reversed by erythropoietin treatment

**Figure 3:**
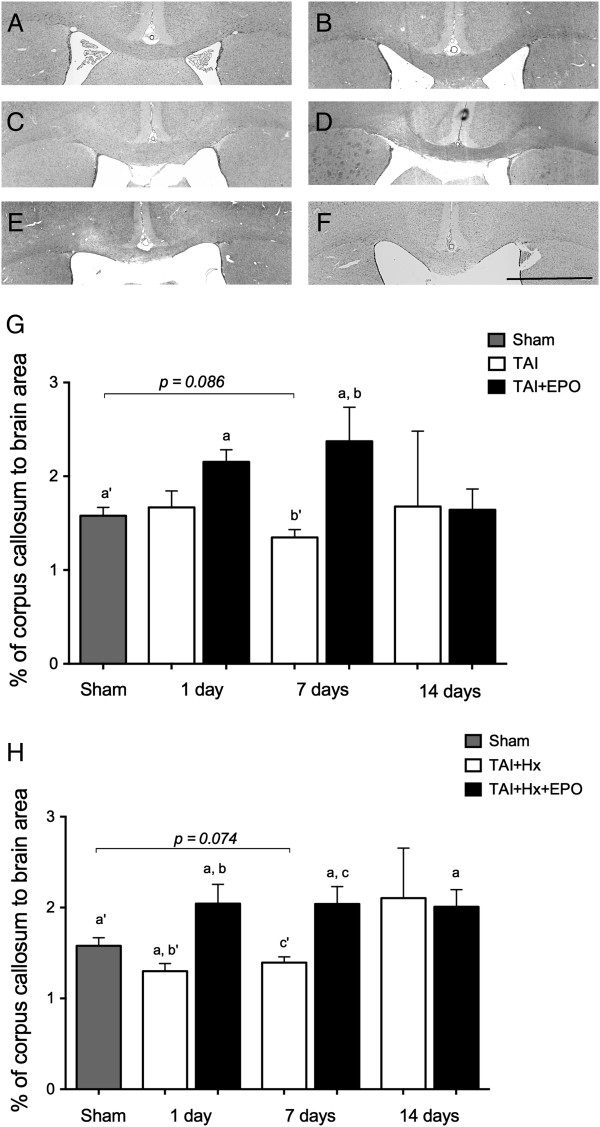
**Atrophy of the corpus callosum is restored with erythropoietin (EPO) therapy in traumatic axonal injury with hypoxia (TAI + Hx) rats.** Atrophy of the corpus callosum after injury was quantified on days 1, 7 and 14 after TAI and TAI + Hx with and without EPO. Photomicrographs taken 1 day postinjury depict treatment effects in the corpus callosum: Sham **(A)** and hypoxia **(B)** rats showed no change in callosal area over the course of 14 days. **(C,G)** There was no change in the measureable area of the corpus callosum after TAI, however there was a significant increase in the callosal area of TAI rats after EPO treatment at 1 and 7 d **(D,G)** when compared to sham, and at 7 d when compared to TAI. **(E,H)** TAI + Hx rats had a significant decrease in the area of the corpus callosum at 1 d when compared to sham. **(F)** With EPO treatment, area of the corpus callosum was significantly increased in TAI + Hx rats at 1 (pictured), 7 and 14 d when compared to vehicle TAI + Hx or sham controls. Data are expressed as mean ± SEM, n = 6/group. Letters matched to letters with apostrophes indicate a significant difference (*P <*0.05 by 2-way ANOVA with post-hoc Bonferroni test). Scale bar = 2 mm.

The corpus callosum is particularly vulnerable to TAI, revealing extensive axonal pathology, which is exacerbated following post-traumatic hypoxia [15]. Therefore, we determined whether there were any changes in the area of the corpus callosum after injury, and whether EPO was able to attenuate any tissue loss. Following sham surgery (Figure 3A), hypoxia (B) and TAI (C,G), the area of the corpus callosum remained unchanged at 1, 7 and 14 days; however TAI rats demonstrated a significant increase in area of the corpus callosum after EPO treatment (D) relative to sham at 1 day (*P <0.05*) and to sham and vehicle treated TAI rats at 7 days (*P <0.05*). A clear effect of TAI + Hx was observed 1 day postinjury compared to sham rats, with the corpus callosum significantly diminished to 82% of sham values (*P <0.05*, two-way ANOVA with Bonferroni post-hoc analysis, Figure 3E,H). When treated with EPO, TAI + Hx rats had a significant 36% increase in callosal area at 1 day compared with vehicle-treated TAI + Hx rats (2.04 ± 0.21 versus 1.3 ± 0.08, in TAI + Hx + EPO versus TAI + Hx, respectively, *P <0.05,* Figure 3F). Interestingly, the area of the corpus callosum of TAI + Hx + EPO rats was also 23% larger compared to sham rats (2.04 ± 0.21 versus 1.58 ± 0.09, *P <0.05*). At 7 d, the reduction in callosal area seen in TAI + Hx rats persisted, with values remaining below that of sham. Conversely, TAI + Hx + EPO rats maintained the integrity of the corpus callosum at 7 days, with values remaining significantly elevated over sham or TAI + Hx (2.04 ± 0.19 versus 1.39 ± 0.06 and 1.58 ± 0.09, TAI + Hx and sham, respectively; *P <0.05*) at 7 days. This increase was still evident at 14 days, where the corpus callosum of TAI + Hx + EPO rats remained significantly larger than sham (*P <0.05*).

### Axonal pathology in the corpus callosum is significantly reduced by erythropoietin in TAI + Hx rats

**Figure 4:**
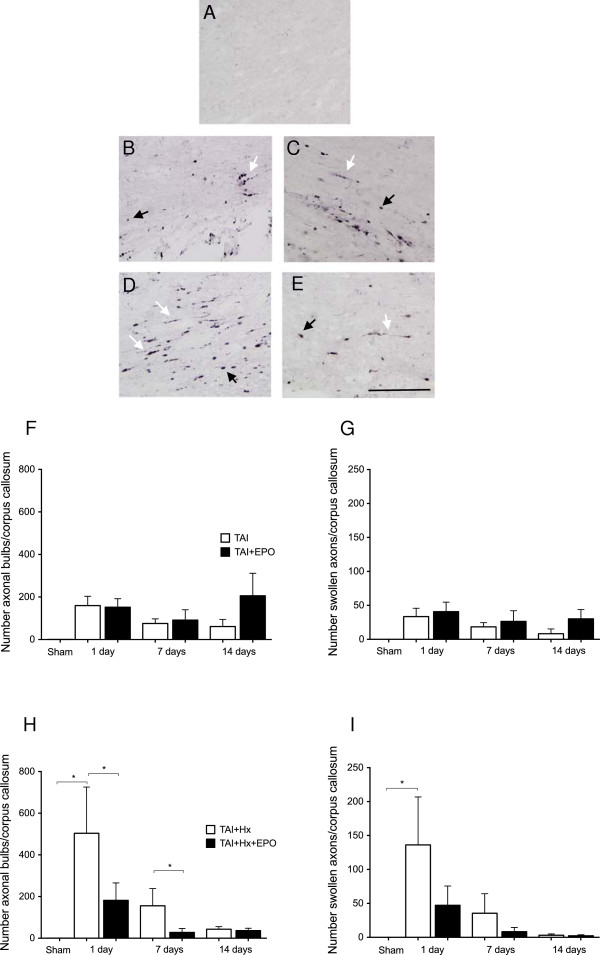
**Axonal pathology in the corpus callosum is significantly reduced by erythropoietin (EPO) after traumatic axonal injury with hypoxia (TAI + Hx).** Representative photomicrographs from the corpus callosum demonstrating axonal bulbs (black arrows) and swollen axons (white arrows) using the NF-200 marker at 1 d following sham **(A)**, TAI **(B)**, or TAI + Hx **(D)** surgery. **(C)** When treated with EPO, TAI rats did not have any reduction in axonal pathology, however TAI + Hx rats **(E)** were afforded remarkable reduction of axonal bulbs when treated with EPO. Scale bar = 100 μm. **(F)** Number of axonal bulbs in the corpus callosum of TAI rats 1, 7 and 14 d postinjury with vehicle or EPO treatment. No benefit of EPO was observed in after TAI. **(G)** Number of swollen axons in the corpus callosum of TAI rats 1,7 and 14 d postinjury with either vehicle or EPO treatment. Again, no differences were observed in swollen axons between TAI or TAI + Hx at any time point. **(H)** Number of axonal bulbs counted in the corpus callosum after TAI + Hx with/without EPO treatment at 1, 7 and 14 d, demonstrating a significant beneficial effect of EPO at 1 and 7 d (*P <0.05; 2-way ANOVA with post-hoc Bonferroni test). **(I)** Although there was a decrease in the number of swollen axons in the corpus callosum of rats allocated to TAI + Hx or TAI + Hx + EPO treatment at 1, 7 or 14 d, this was not statistically significant. Data shown as mean ± SEM, n = 6 per group per time point.

Axonal damage was assessed using the NF-200 heavy-chain marker, which highlights the gross pathology of diffuse axonal injury in the form of swollen axons and terminal bulb formation [40]. In sham (Figure 4A) and hypoxia animals (not shown), scant NF-200 positive staining was observed at all time points, with almost no positive bulbs or axons counted after surgery.

Generally, axonal damage after TAI or TAI + Hx was similar to that we previously reported in this combined insult model with respect to regional distribution through the white matter and achieving a particular prominence in the corpus callosum and pyramidal tracts of the brainstem [15]. Axonal pathology was most striking in TAI and TAI + Hx groups 1 day postinjury (Figure 4B,D, respectively), with the presence of both spherical terminal axonal bulbs and long, tortuous axons.

#### Axonal pathology in the corpus callosum

In the corpus callosum, the largest numbers of axonal bulbs counted in each section were observed acutely at 1 day after TAI (159.6 ± 43.99; Figure 4F), with numbers of bulbs gradually decreasing at 7 days (75.7 ± 21.69) and remaining constant until 14 days (61.0 ± 33.65), with a significant effect of time overall observed by two-way ANOVA (*P <0.01*).

In TAI rats treated with EPO, numbers of axonal bulbs were similar to those observed in vehicle-treated rats at 1 and 7 days (152.10 ± 39.96 and 91.47 ± 48.56, respectively; Figure 4C). Interestingly, at 14 days after TAI with EPO treatment, there was an increase (though not significant) in the numbers of axonal bulbs when compared to either TAI rats at 14 days, or TAI + EPO rats at 7 days (205.7 ± 105.9 versus 61.0 ± 33.65 or 91.47 ± 48.56, respectively).

In TAI rats, numbers of swollen axons were also highest at 1 day, with similar numbers detected irrespective of vehicle or EPO treatment (33.48 ± 12.32 versus 40.75 ± 14.07 in TAI versus TAI + EPO, Figure 4G). While there was a gradual decline in numbers of swollen axons in TAI rats over the course of 2 weeks, EPO treatment only resulted in a slight decrease in axonal damage at 7 days, persisting out to 14 days (30.22 ± 13.64 versus 8.313 ± 7.04, TAI + EPO versus TAI at 14 days). Overall, there was no statistically significant difference in axonal pathology with respect to either axonal bulbs or swollen axons detected between TAI and TAI + EPO rats at any time point examined.

With a substantially more severe injury caused by additional post-traumatic hypoxia, TAI + Hx rats suffered a greater burden of axonal damage, particularly in the form of axonal bulbs, which were detected more than three- fold higher than that observed in TAI alone on day 1. Similar to TAI animals, axonal bulbs were most prominent at 1 day after TAI + Hx injury (Figure 4H) with a decline by approximately two-thirds by 7 days, and by two-thirds again by 14 days (503.6 ± 222, 155.5 ± 82.77, 43.19 ± 12.21 at 1, 7 and 14 days, respectively). Overall, there was a significant effect of EPO treatment on minimising the number of axonal bulbs over the time course examined (*P <0.01* by two-way ANOVA), with the most prominent reduction of axonal bulbs of 64% by EPO observed at 1 day after TAI + Hx (503.6 ± 222.0 versus 181.8 ± 83.54 for TAI + Hx versus TAI + HX + EPO, respectively, *P <0.05*). A significant decrease of 83% was also seen at 7 days after injury, when axonal pathology was largely diminished in TAI + Hx + EPO rats, while it persisted in TAI + Hx rats (155.5 ± 82.77 versus 27.76 ± 18.83, *P <0.05*). However, at 14 days, numbers of axonal bulbs were similar in both TAI + Hx and TAI + Hx + EPO rats, with no significant difference observed between the two groups (43.19 ± 12.21 versus 36.21 ± 11.46, *P = 0.68*).

Examination of axonal swelling via NF-200 staining in TAI + Hx rats also demonstrated abundant pathology throughout the corpus callosum, and although maximal at 1 day postinjury, the numbers of swollen axons were substantially less when compared to numbers of axonal bulbs. EPO treatment reduced the numbers of swollen axons by 65% (136.1 ± 70.65 versus 47.14 ± 28.46; Figure 4I) though this effect was not statistically significant. TAI + Hx + EPO rats also experienced a decrease in axonal swelling at 7 days, with a reduction of 77% compared to vehicle (35.43 ± 28.87 versus 8.26 ± 6.13), though again this was not statistically significant. At 14 days, axonal pathology was largely diminished in both TAI + Hx and TAI + Hx + EPO groups, with scant numbers of damaged axons detected.

#### Axonal pathology in the brainstem

In the brainstem, analysis of NF-200 immunohistochemistry revealed extensive axonal pathology, particularly with respect to the pyramidal tracts in which abundant axonal bulbs and swollen axons were detected, mostly at 1 day postinjury. In keeping with findings in the corpus callosum, no axonal pathology was detected in sham or hypoxia only animals at any time point examined.

**Figure 5:**
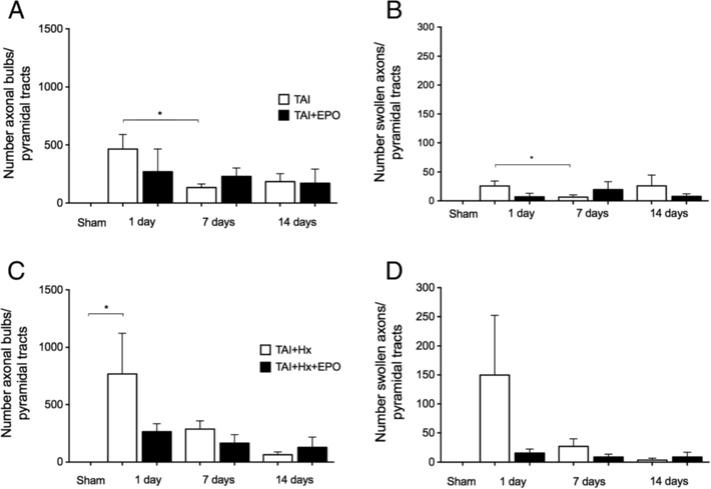
**Erythropoietin** (E**PO) attenuates axonal pathology in the pyramidal tracts of the brainstem. (A)** Axonal bulbs in the pyramidal tracts of the brainstem in traumatic axonal injury (TAI) rats with/without EPO treatment at 1, 7 or 14 d. No statistical difference was observed at any time point amongst the treatment groups, however there was a spontaneous decrease in the number of axonal bulbs at 7 d in TAI rats only. **(B)** Number of swollen axons in the pyramidal tracts of TAI and TAI + EPO rats at 1, 7 and 14 d. EPO treatment failed to reduce axonal swelling, however there was an overall decrease in the number of swollen axons in TAI rats at 7 d. TAI + Hx rats had a strikingly more axonal bulbs **(C)** and swollen axons **(D)** in the pyramidal tracts of the brainstem, with both substantially (though not significantly) reduced at 1 d by treatment with EPO. Data shown as mean ± SEM, n = 6 per group per time point.(*P <0.05, 2-way ANOVA with post-hoc Bonferroni test).

TAI rats had significantly more axonal bulbs in the pyramidal tracts when compared to the corpus callosum. This was detected particularly at 1 day postinjury, where there was an increase of 66% in the pyramidal tracts (466.3 ± 123.5 versus 33.48 ± 12.32, *P <0.05*). The number of axonal bulbs in TAI rats decreased over 7 days (466.3 ± 123.5 versus 133.4 ± 31.26, *P <0.05*), to remain constant until 14 days (185.50 ± 68.98; Figure 5A). In EPO treated TAI rats, a small, nonsignificant decrease in the number of axonal bulbs was observed at 1 day, relative to TAI alone (466.3 ± 123.5 versus 270.5 ± 196.2, *P = 0.43*), with the number of bulbs remaining unchanged over the course of 14 days.

The number of swollen axons observed in TAI rats was highest in the brainstem at 1 day (Figure 5B). The number of swollen axons was significantly decreased over time in the pyramidal tracts of TAI rats at 7 day (25.78 ± 8.53 versus 6.52 ± 3.87, *P <0.05*), with relatively similar numbers of swollen axons observed at 14 day. EPO treatment did not show any effect in reducing the number of swollen axons at any time point examined when administered after TAI.

TAI + Hx rats also showed the highest number of axonal bulbs in the brainstem at 1 day post-injury, with a decrease of 62% at 7 days (to 286.70 ± 71.90), and a further decrease by 78% at 14 days (to 64.40 ± 24.55; Figure 5C), though this was not statistically significant. When treated with EPO, TAI + Hx rats had a decrease in number of NF-200 positive axonal bulbs of 64%, at 1 day postinjury, however this was not statistically significant (767.8 ± 353.4 versus 263.3 ± 70.17, *P = 0.21*).

TAI + Hx + EPO rats also had a marked decrease in the number of swollen axons at 1 day in the pyramidal tracts when compared to untreated TAI + Hx rats (Figure 5D) and although not statistically significant, the mean reduction of axonal swellings exceeded 90% (149.8 ± 102.4 versus 15.62 ± 6.892, *P = 0.94*). The number of swollen axons was substantially decreased in vehicle TAI + Hx rats at 7 days, with a further decline at 14 days, while the number of swollen axons remained relatively constant in TAI + Hx + EPO rats up to 14 days.

### Dendritic loss in TAI + Hx rats is largely rescued by erythropoietin therapy

**Figure 6:**
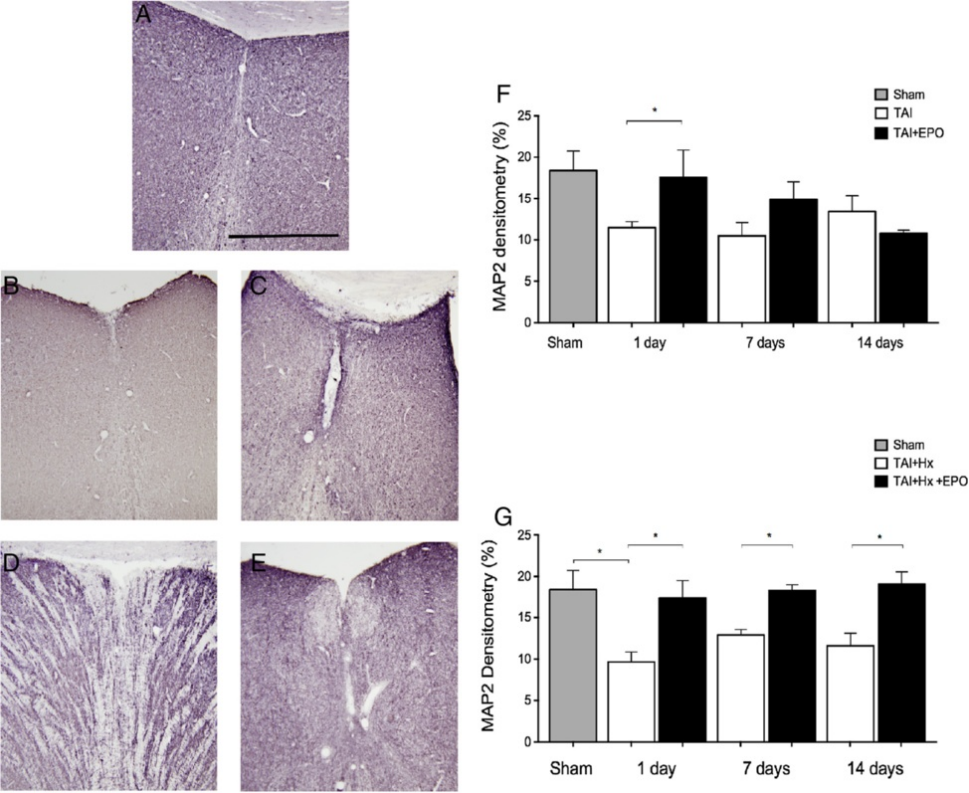
**Loss of dendrites is regionally variable in traumatic axonal injury (TAI) and traumatic axonal injury with hypoxia (TAI + Hx) rats, and is largely rescued by erythropoietin (EPO). (A)** Representative photomicrograph of dense MAP2 immunoreactivity observed in the lateral septal nucleus of a sham-treated rat. **(B)** 1 d after TAI, there was a diffuse loss of MAP2 staining, although the cytoarchitecture remained similar to sham. **(C)** EPO treatment improved the appearance of MAP2 reactivity in the lateral septal nu. **(D)** Striking changes in MAP2 staining were observed in TAI + Hx rats, in which changes both in the intensity of MAP2 staining with regions completely devoid of MAP2 immunoreactivity. **(E)** EPO markedly rescued dendritic loss in TAI + Hx + EPO rats. Scale bar = 100 μm. **(F)** Densitometric analysis of TAI rats revealed a decrease in MAP2 labeling at 1 d compared to sham, and was significantly improved by EPO treatment. **(G)** TAI + Hx rats had a more substantial loss of MAP2 1 d postinjury, and EPO treatment significantly restored MAP2 density to sham levels. With this protective effect maintained at 7 and 14 d. Data shown as mean ± SEM, n = 6 per group per time point. *P <0.05, 2-way ANOVA with post-hoc Bonferroni test.

Dense MAP2 immunoreactivity was found throughout the brains of sham animals localised to the soma and proximal dendrites of neurons, with scant expression in the white matter (Figure 6A). In TAI rats, MAP2 immunohistochemistry revealed a diffuse loss in staining, although the cytoarchitecture was maintained in the neuronal soma and dendrites (Figure 6B). Using densitometric analysis, the reduction in MAP2 was most prominent at 1 day in the lateral septal nucleus of TAI rats when compared to sham using densitometric analysis (11.47 ± 0.72 versus 18.4 ± 2.33, *P <0.05*; Figure 6F). When TAI rats were treated with EPO, a significant preservation of MAP2 density was observed at 1 day (17.59 ± 3.27 versus 11.47 ± 0.72, *P <0.05* TAI + EPO versus TAI), with values similar to sham levels (Figure 6C,F). TAI rats showed no change in MAP2 density over time (up to 14 days), while interestingly, TAI + EPO rats had a small, though non-significant decrease of 40% in MAP2 density over the course of 14 days.

TAI + Hx rats demonstrated remarkable changes both in the intensity of MAP2 staining and the integrity of the cytoarchitecture, with large regions of the lateral septal nucleus completely devoid of MAP2 immunoreactivity in a ‘stripe-like’ pattern at 1 day postinjury (Figure 6D), at which time there was a significant decrease to approximately half of the MAP2 density observed for sham animals (9.64 ± 1.2 versus 18.4 ± 2.33, *P <0.05*, Figure 6G). At 7 days, there was a small recovery in MAP2 loss in TAI + Hx rats, with a trend to significance (9.64 ± 1.20 versus 12.90 ± 0.69, *P = 0.06*; Figure 6G). At 14 days, values were comparable to those observed at 7 days, with no further recovery in MAP2 density in vehicle TAI + Hx rats. TAI + Hx rats treated with EPO showed the most striking recovery of MAP2 density at 1 day (9.64 ± 1.20 versus 17.33 ± 2.16, *P <0.05*; Figure 6E,G), with values observed significantly higher than TAI + Hx, and similar to sham. This effect of EPO in maintaining MAP2 density was also observed at 7 and 14 days, with values in TAI + Hx + EPO rats remaining similar to sham and significantly higher than vehicle-treated groups at every time point examined.

### Erythropoietin administration enhances the expression of erythropoietin receptor

Baseline cellular expression of the EpoR was found throughout the brains of sham and hypoxia animals, with no obvious specificity in distinct brain regions. In injured animals, EpoR expression was enhanced throughout the white matter, overlapping with axonal damage in the pyramidal tracts of the brainstem and the corpus callosum, in which positively stained cells appeared to be mostly small and membrane-bound.

#### EpoR expression in the corpus callosum

**Figure 7:**
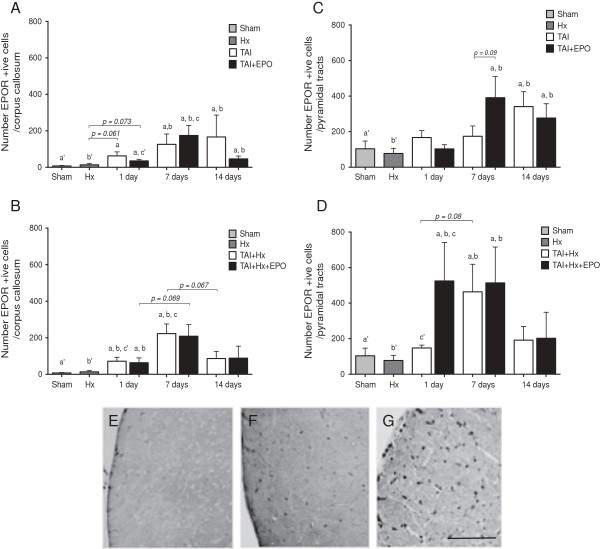
**Erythropoietin (EPO) administration enhances the expression of the erythropoietin receptor (EpoR) in the brainstem of traumatic axonal injury with hypoxia (TAI + Hx) rats. (A)** In the corpus callosum, TAI rats had a significant elevation in EpoR numbers over sham at 1,7 and 14 days, with similar numbers observed in TAI + EPO rats over this time course. **(B)** TAI + Hx and TAI + Hx + EPO treatments both resulted in increased numbers of positive cells at 1 day compared to sham, with both groups peaking at 7 days before returning to sham levels at 14 days. **(C)** TAI and TAI + EPO rats showed a similar expression pattern of EpoR in the brainstem at 1 d, however at 7 d, TAI + EPO rats had a substantial increase in the number of cells staining positively for EpoR. **(D)** No increase in EpoR positive cells was observed in the pyramidal tracts at 1 d after TAI + Hx; however in these rats, EPO administration induced a significant increase in EpoR expression. At 7 d, the number of EpoR-positive cells increased in TAI + Hx similarly to TAI + Hx + EPO. Letters matched to letter with apostrophe indicate a significant difference, *P* <0.05; 2-way ANOVA with post-hoc Bonferroni test. Data expressed as mean ± SEM, n = 6/group. *(E) Photomicrograph demonstrating low level of EpoR expression in the pyramidal tracts after sham surgery, a mild increase in TAI + Hx **(F)**, and a significant increase in EpoR-positive cells in TAI + Hx + EPO rats **(G)**. Scale bar = 100 μm.

In the corpus callosum, TAI rats had a significant increase in the number of cells expressing the EpoR at 1 day postinjury (62.64 ± 22.11 versus 7.42 ± 2.34, *P <0.05*, Figure 7A), which increased further, albeit nonsignificantly at 7, and at again at 14 days relative to 1 day, to reach a peak of 166.40 ± 120.20 EpoR positive cells. With EPO treatment, the number of EpoR positive cells was slightly but significantly higher over sham at 1 day post-injury, and further increased significantly to peak at 7 days (174 ± 55.13 versus 35.53 ± 8.28, TAI + EPO 7 days versus 1 day, respectively; *P <0.05*). EpoR expression subsided at 14 days in TAI + EPO rats, with values similar to those observed at 1 day post-injury.

At 1 day postinjury, TAI + Hx rats had a significant increase in number of cells expressing the EpoR in the corpus callosum (Figure 7B, *P <0.05*), with a comparable number of cells to TAI rats at this time. TAI + Hx rats experienced a peak in the numbers of cells expressing EpoR at 7 days, with a significant increase from 1 day (223.0 ± 52.55 versus 71.81 ± 21.74, *P <0.05*). In these rats, EpoR expression had decreased by 14 days, with similar numbers to 1 day. Regardless of whether rats had vehicle or EPO treatment, an almost identical pattern of EpoR expression was obtained in the corpus callosum of all TAI + Hx rats, displaying a peak at 7 days.

#### EpoR expression in the brainstem

Examination of EpoR staining in the brainstem revealed increased expression of EpoR across all groups compared to the corpus callosum, regardless of treatment.

Sham and hypoxia-only rats had a comparable basal expression of EpoR in the pyramidal tracts of the brainstem at 1 day (Figure 7E). At day 1, TAI rats revealed a small, non-significant increase in the number of cells expressing the EpoR (Figure 7C) when compared to either sham or hypoxia treatments. Interestingly, at 7 days the number of cells expressing EpoR remained similar to that observed at day 1 in the pyramidal tracts, and a peak in EpoR expression was delayed to 14 days postinjury, with double the number of EpoR positive cells when compared to TAI rats at 7 days, sham or hypoxia rats (341.10 ± 83.52 (TAI 14 days) versus 174 ± 58.23 (TAI 7 days), 103.80 ± 43.24 (sham), or 77.67 ± 28.97 (hypoxia); *P <0.05* TAI 14 days versus sham, hypoxia only, Figure 7C). Upon EPO administration, TAI rats did not show increased number of cells expressing the EpoR at 1 day; however at 7 days, there was a substantial increase of EpoR positive cells, with a statistical trend for increase over TAI vehicle rats (391.0 ± 118.70 versus 174 ± 58.23, *P = 0.09*). By 14 days, this number had slightly decreased and was akin to peak values observed for vehicle TAI rats, though it remained significantly higher when compared to sham or hypoxia rats (*P <0.05*).

In TAI + Hx rats at 1 day postinjury, there was a similar level of EpoR expression compared to sham, hypoxia and TAI rats, with no discernable increase in positive cells (Figure 7D,F). However, at day 7, a sudden increase in EpoR expression occurred, reaching a peak value greater than at 1 day (463.60 ± 155.10 versus 147.80 ± 16.18, TAI + Hx 7 days versus 1 day, *P = 0.05; P <0.05,* TAI + Hx 7 days versus sham), with a reduction to control levels at 14 days.

Interestingly, upregulation of EpoR in TAI + Hx + EPO rats occurred earlier, at 1 day postinjury (Figure 7G), with the number of EpoR positive cells being significantly higher than those seen in sham, hypoxia, TAI or TAI + Hx groups (*P <0.05* to all groups, Figure 7D). This increased EpoR expression was maintained in TAI + Hx + EPO rats at 7 days, while by 14 days, numbers of EpoR positive cells in TAI + Hx + EPO rats declined to values similar to those of TAI + Hx rats.

### The erythropoietin receptor is expressed predominantly on neurons

**Figure 8:**
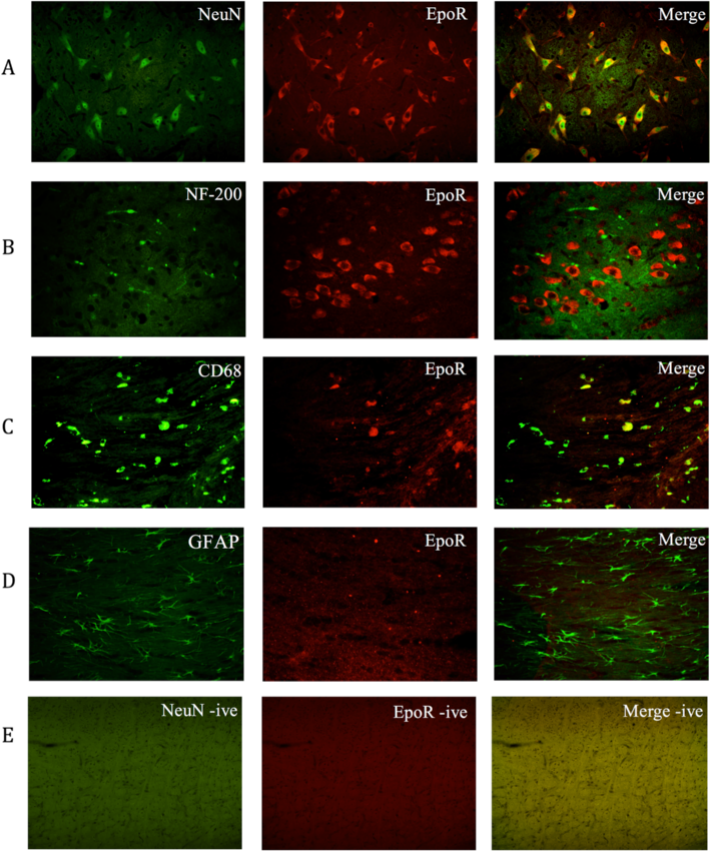
**The erythropoietin receptor** (**EpoR) is expressed predominantly on neurons**. Representative images of immunofluorescent double labeling employed to determine co-localisation between EpoR (middle panel) and neurons (**A**; NeuN), damaged axons (**B**; NF-200) in the brainstem at 1d, macrophages/microglia (**C**; CD68), and astrocytes (**D**; GFAP) in the corpus callosum at 7 d. EpoR co-localised mostly with NeuN, having a sporadic overlap with CD68. No colocalisation was found for EpoR and NF-200 or GFAP. **(E)** Demonstrates a lack of positive staining in negative control slides. Scale bar = 100 *μ*m.

To determine the type of cells expressing EpoR, immunofluorescent double labelling was employed on brain sections at various times in combination with markers for neurons (NeuN), damaged axons (NF-200), macrophages/microglia (CD68) and astrocytes (GFAP). In the pyramidal tracts, there was a strong co-localisation between EpoR and NeuN at 1 day post-TAI (Figure 8 panel A), with high expression in neurons throughout the brain regardless of treatment. No overlap was seen between EpoR and NF-200 staining at 1 (pictured) or 7 days post-TAI, confirming that EpoR was not expressed on damaged axons (8B). CD68 staining for macrophages and microglia revealed a partial colocalisation with EpoR staining at 7 days in a small percentage of CD68 positive cells distributed in the corpus callosum (8C). Examination of GFAP staining 1 day postinjury showed no overlap with EpoR in the corpus callosum (8D) or any other region of the brain. To ensure the staining visualised was specific, appropriate controls were carried out omitting the primary antibody for each marker. Panel E demonstrates a lack of positive staining in these negative control slides (8E).

### Accumulation of CD68 positive macrophages and microglia is reduced by erythropoietin treatment after TAI + Hx

**Figure 9:**
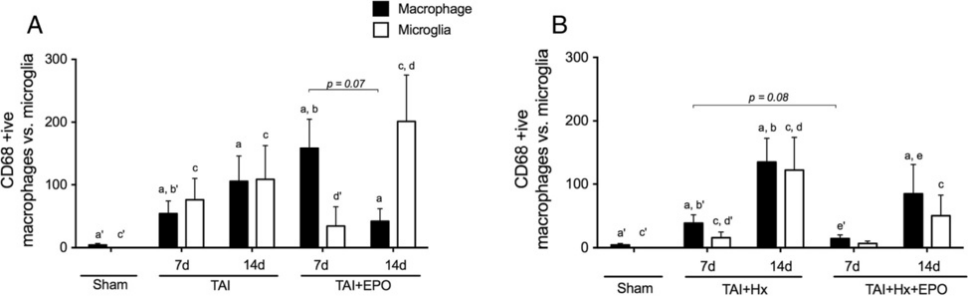
**Accumulation of CD68-positive macrophages and microglia is reduced by erythropoietin (EPO) treatment after traumatic axonal injury with hypoxia (TAI + Hx).** In the corpus callosum CD68-positive (+ive) staining was classified on the basis of macrophage or microglial appearance, with the number of each counted separately. **(A)** The ratio of macrophages to microglia demonstrated a shift between 7 and 14 d in TAI + EPO rats, with an early predominance of macrophages, followed by a more abundant accumulation of microglia at 14 d. **(B)** The ratio of macrophages to microglia was similar at both 7 and 14 days in both TAI + Hx and TAI + Hx + EPO rats. Data expressed as mean ± SEM, n = 6/group.

Analysis of brain sections from sham and hypoxia rats revealed low numbers of CD68 positive cells throughout the brain at each time point examined. At 7 days postinjury, there was a marked increase in accumulation of CD68 positive cells in TAI brains which was most prominent in the corpus callosum, concomitant to axonal damage. CD68 positive cells were classified and quantified as being of a microglial or macrophage appearance based on the presence/absence of processes and amoeboid morphology as previously described [15]. At 7 days post TAI, rats had similar numbers of CD68 positive macrophages and microglia, with both significantly elevated when compared to sham (Figure 9A, *P <0.05*). At 14 days, the numbers of both cell types increased slightly but non-significantly (*P = 0.34* for macrophages and *P = 0.65* for microglia) and maintained a relative constant ratio to each other, to reach peak numbers of 105.50 ± 40.24 (macrophages) and 108.60 ± 54.19 (microglia), respectively.

Interestingly, while CD68 positive macrophages were highest in number in the corpus callosum of TAI rats at 14 days, EPO treatment resulted in an earlier peak in macrophage accumulation at 7 days, with counts significantly higher than those observed for vehicle-treated rats *P <0.05*; Figure 9A). This relationship was inverse at 14 days, at which time EPO-treated TAI rats had a sharp decrease in macrophages to approximately one-third, although this did not reach statistical significance (*P = 0.07*). While numbers of macrophages decreased with EPO treatment at 14 days, numbers of microglia increased significantly by almost six times relative to numbers in TAI + EPO rats at 7 days (201.30 ± 73.68 versus 34.27 ± 30.59, *P <0.05*).

Similar to TAI, TAI + Hx rats had an abundant accumulation of both macrophages and microglia in the corpus callosum at 7 days and 14 days postinjury (Figure 9B). EPO treatment decreased numbers of macrophages at 7 days when compared to vehicle-treated TAI + Hx rats, although not significantly (14.24 ± 5.81 versus 38.70 ± 13.03, *P = 0.08*). At day 14, the number of macrophages in TAI + Hx + EPO rats increased significantly compared to day 7 (*P <0.05*). EPO treatment caused a small, non-significant decrease in macrophages when compared to TAI + Hx. In regard to the number of microglia, a reduction of more than 60% was found in TAI + Hx + EPO rats when compared to vehicle TAI + Hx rats at 7 days, though this was not statistically significant (6.58 ± 3.75 versus 15.66 ± 0.03, *P = 0.42*). Likewise, at 14 days, numbers of microglia in TAI + Hx + EPO rats (50.17 ± 32.5) were substantially reduced by 60% compared to vehicle TAI + Hx rats (122.10 ± 51.94, *P = 0.38*).

### IL-1β is enhanced after TAI + Hx, and markedly suppressed by erythropoietin

**Figure 10:**
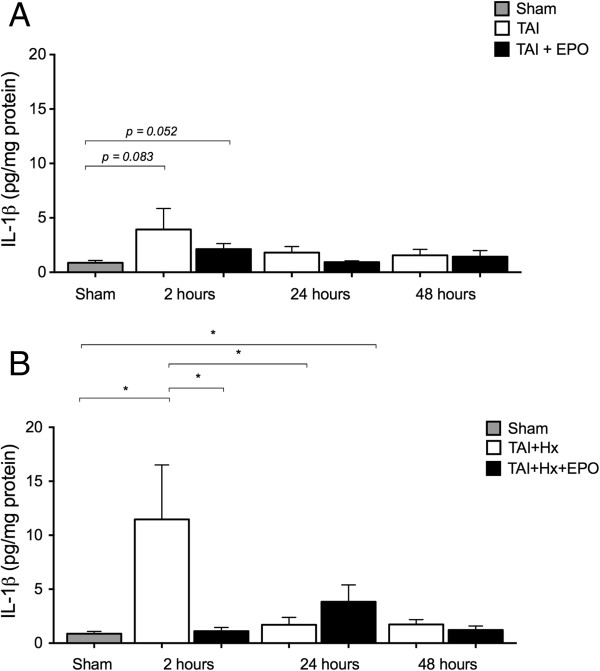
**The proinflammatory cytokine IL-1β is upregulated acutely after traumatic axonal injury with hypoxia (TAI + Hx), and its production is suppressed acutely by erythropoietin (EPO). (A)** IL-1β levels were elevated acutely above sham levels at 2 h after TAI, with an increase approaching significance to sham in both TAI and TAI + EPO groups. No statistical differences were found between TAI and TAI + EPO groups at any time point examined (2, 24, or 48 h). **(B)** TAI + Hx rats had a striking significant increase in IL-1β levels acutely at 2 h when compared with sham. When TAI + Hx rats were treated with a single dose of EPO 1 h after injury, IL-1β levels were suppressed and remained at sham values, in contrast to saline-treated TAI + Hx rats. Data expressed as mean ± SEM, n = 4/group. *P <0.05; 2-way ANOVA with post-hoc Bonferroni test.

While IL-1β levels were elevated by 78% 2 hours after TAI (Figure 10A), this increase was not significant relative to sham control (3.935 ± 1.926 versus 0.5370 ± 0.20, *P = 0.08*). After this time, TAI rats showed a steady decrease in IL-1β, with a 48 hour concentration of 1.55 ± 0.55 pg/mg protein, which although higher (0.87 ± 0.21 pg/mg protein) was not statistically different to sham. Conversely, TAI + Hx rats had a sharp significant increase in IL-1β levels at 2 hours relative to sham (11.47 ± 5.05 versus 0.87 ± 0.21 pg/mg protein, *P <0.05*; Figure 10B); which was an almost three-fold increase when compared to TAI. Interestingly, the upregulation of IL-1β in the white matter was substantially higher compared to the concentration detected in the cortex of TAI-Hx rats in our previously published study (3.10 ± 0.56 pg/mg protein) (Yan *et al.* 2011). IL-1β levels dropped markedly from 2 hours to 24 hours (11.47 ± 5.05 versus 1.70 ± 0.68 pg/mg protein, *P <0.05*), with values returning to sham values at both 24 and 48 hours postinjury.

When TAI rats were given EPO, a trend toward increase in IL-1β levels was detected at 2 hours relative to sham (*P = 0.052*), although interestingly, the level of IL-1β was slightly (but not significantly) lower than that observed for vehicle treated rats. At 24 hours, IL-1β levels returned to sham values, and remained similar until 48 hours. Comparison of IL-1β concentrations between TAI and TAI + EPO groups showed no statistical changes at any time point examined (2, 24, or 48 hours).

Conversely, in TAI + Hx rats, a single dose of EPO given 1 hour after injury was sufficient to keep IL-1β production to sham levels at 2 hours, with a dramatic significant decrease from vehicle-treated TAI + Hx rats of more than 90% (11.47 ± 5.05 versus 1.11 ± 0.34 pg/mg protein, *P <0.05*). Interestingly IL-1β levels in TAI + Hx + EPO rats were significantly higher at 24 hours when compared to sham (3.83 ± 1.57 pg/mg protein, *P <0.05*).

## Discussion

In this study, we have demonstrated for the first time the efficacy of EPO in reducing sensorimotor and cognitive deficits, axonal and dendritic pathology, and the acute inflammatory response, concomitant to an increased expression of the endogenous EpoR in a combined rat model of diffuse brain injury and post-traumatic hypoxia. Most importantly, these benefits of EPO treatment did not occur in animals subjected to TAI alone, suggesting that the presence of hypoxia after injury has bolstered the neuroprotective capacity of EPO, in a manner not seen in normoxic conditions of TAI.

EPO has been shown to improve sensorimotor and cognitive deficits in rats after both CCI and cryogenic lesion injuries [22-24,41], with a therapeutic window of administration up to 24 h after injury reported to provide behavioural benefit [35]. In keeping with the literature, in our study the injection of EPO at 1 and 24 h after TAI + Hx resulted in a sustained improvement in the Rotarod. This test is an efficient measure of sensorimotor impairment, which highlights fine changes in vestibulomotor ability as well as prolonged deficits that may be overlooked by less sensitive sensorimotor tests [36]. EPO-induced amelioration of motor function after TAI + Hx on the Rotarod occurred from 5 days postinjury, with this beneficial effect maintained to 14 days. While it is difficult to identify in a diffuse injury model the precise area of the injured brain causing functional deficit, and pinpoint the mechanism through which the multifunctional EPO is able to provide benefit, there are several possibilities. The most frequently posited hypothesis attributes EPO-mediated neurological improvement to a greater cerebral oxygenation and subsequent mitigation of metabolic dysfunction, as observed both clinically and experimentally after ischemia and subarachnoid haemorrhage [42-44]. However, a recent study by Meng and colleagues reported that EPO’s therapeutic effects occur independently of increased blood cell volume [35], a finding also supported in the context of retinal degeneration, in which tissue improvement was detected without a concomitant increase in haematocrit [45]. Of particular note is the finding that no beneficial effect of EPO was observed in sensorimotor or cognitive tasks when administered to TAI animals, indicating that the benefit of EPO is intricately bolstered by hypoxia. In support of this theory are the findings that EPO enhances neurological recovery in models of hypoxia-ischemia, particularly with respect to sensorimotor and spatial deficit [46-48], suggesting that hypoxia induction is of critical importance in the potentiation of the EPO-mediated response after brain insults. Further, the oxygen sensing molecule hypoxia inducible factor 1 (HIF-1α) is stabilised only under hypoxic conditions by binding to the EPO promoter to upregulate downstream target genes including *EPO* and *EPOR*[49]. This supports an intimate relationship between hypoxia, EPO, and expression of the EPO receptor and therapeutic efficacy of EPO [50,51].

Many long-term behavioural consequences of TBI have been attributed to circuit dysfunction as a result of white matter damage and degeneration [52]. This degeneration may be exacerbated by hypoperfusion in an acute and chronic setting, both experimentally [53-55] as well as clinically, with studies reporting haemodynamic abnormalities and prolonged hypoperfusion up to 6 years after TBI [56,57]. A key factor for cognitive recovery is the improvement in white matter blood flow [56]; so it may be speculated that EPO administration does ameliorate cerebral blood flow dysfunction, leading to an overall improvement in a cognitive context. Here, we have observed a significant atrophy of the corpus callosum in TAI + Hx rats, with this pathology persisting to 7 days at which time we detected severe cognitive dysfunction in the NORT. Interestingly, we found a favourable effect of EPO in reversing the degradation of the corpus callosum at each time point examined, and at the same time, a significant improvement to sham levels in the NORT, indicating that cognitive function may indeed be linked to white matter damage and degeneration. At the same time, we found that not only did EPO significantly preserve the integrity of the corpus callosum in TAI + Hx + EPO rats when compared to TAI + Hx rats, but it also significantly enhanced the callosal area when compared to sham treated rats.

Of particular interest for diffuse brain injury, we observed a highly significant reduction in the number of terminal axonal bulbs in the corpus callosum at 1 day using the cytoskeletal protein marker NF-200 in TAI + Hx + EPO rats when compared to TAI + Hx, indicating that EPO was able to prevent progression of secondary axonal pathology to a terminal state. Because EPO was given at a first dose 1 hour after TAI + Hx and not prior to injury, this reduction in axonal bulbs can be attributed only to EPO’s ability to mediate secondary pathological events, with no effect on any potential immediate shearing of axons. Interestingly, we also observed a decrease in the number of swollen axons present in the corpus callosum at 1 day, and although this decrease was not significant, perhaps due to variability observed within groups, there was still a mean decrease of almost three-fold when compared to TAI + Hx rats, indicating that EPO was able to substantially reduce axonal swelling by mediation of cytoskeletal degeneration/disruption, potentially through attenuation of calpain-mediated spectrin proteolysis, as has been shown when applied in a hippocampal slice model of oxygen-glucose depravation [58]. At 7 days, TAI + Hx + EPO rats maintained a lower number of both axonal bulbs and swollen axons, indicating that EPO administration acutely at 1 and 24 hours after injury was sufficient not only to reduce acute secondary events, but also to prevent progression of pathology with a terminal end-point. These findings were echoed in the pyramidal tracts of the brainstem, and although we found that the reduction in number of bulbs and swollen axons was not significant (again likely due to intragroup variability) EPO apparently exerted a beneficial effect in reducing pathological damage to the axonal cytoskeleton in this region.

The microtubule-associated protein MAP2 is essential for the function of the somatodendritic compartment of neurons, and is the most abundant cytoskeletal protein in the brain [59]. MAP2 has been shown to be particularly vulnerable to brain injury, with a rapid loss of staining, which reveals significant dendritic pathology. In focal injuries such as CCI, MAP2 loss is most prominent in proximity to the lesion site [60] and is closely associated with cell death, whereas in FPI, its reduction is regionally specific, with the hippocampus showing stronger loss, and the cortex largely spared [61]. In this study, we found a regional variation in MAP2 loss, with the caudate putamen displaying most evident disruption. Photomicrographs of this most damaged region after TAI + Hx revealed patches devoid of MAP2 reactivity, with densitometric analysis showing a decrease in density to approximately half of that observed in sham rats. Importantly, this alteration persisted until 14 days, indicating that while cytoskeletal and transport dysfunction in the axon have resolved, ongoing dendritic damage may contribute to prolonged pathophysiology observed after diffuse injury, and failure to recover from functional deficits [61]. EPO administration to TAI + Hx rats prevented injury-induced MAP2 loss as early as 1 d, after a single injection of EPO given at 1 hour post-injury. This beneficial effect of EPO was also maintained at 7 and 14 days, with no differences detectable from sham levels. The dendritic sparing afforded by EPO may also contribute to behavioural improvement, with early dendritic protection leading to an overall recovery in functionality. Loss of MAP2 has been postulated to be due to proteolytic degradation by Ca^2+^-dependent proteases such as calpain, which, as mentioned, is also thought to be a main perpetrator of spectrin damage in the axon. This finding of decreased MAP2 loss in conjunction with reduced NF-200 positive staining indicates that EPO’s beneficial effects on the neuron may be due to a reduction in Ca^2+^ influx, and/or calpain-mediated pathology. Interestingly, calpain also degrades the membrane-bound EpoR, with this effect reversed by calpain inhibition [62]. Therefore, EPO’s inhibition of calpain activity might also exert effects on survival of the EpoR, and thus positively modulate its ability to bind to available EpoRs.

While there is a low constitutive expression of neuronal EpoR in the normal rodent brain [19,63,64], the number of cells expressing EpoR has been shown to increase dramatically in response to hypoxia/ischemia clinically [65,66] and experimentally [67-71]. EpoR is also rapidly upregulated in experimental stroke and global ischemia [19,72,73] and focal TBI [74,75], although upregulation of endogenous Epo/EpoR *per se* is not sufficient for neuroprotection [76]. Increased expression of EpoR does, however, provide a platform for exogenous EPO treatment, which has also been shown to increase EpoR expression further [76-78]. As the level of EpoR expression is thought to mediate the neuroprotective effects of EPO [79], we were interested to determine whether hypoxia alone, TAI, or the combination of both insults would upregulate the expression of the EpoR, and whether exogenous EPO administration would further boost this expression. We demonstrated that the highest expression of EpoR was in the pyramidal tracts of the brainstem, and while we found a small, nonsignificant increase in the number of EpoR positive cells after TAI or TAI + Hx at 1 day, EPO administration significantly bolstered the number of cells staining positively for the EpoR in TAI + Hx rats when compared to their vehicle treated counterparts. In addition, EpoR levels remained significantly high in these TAI + Hx + EPO rats at 7 days, indicating a long-lasting stimulation of EpoR expression by exogenous EPO.

Intriguingly, while we saw a beneficial effect of EPO in boosting EpoR expression in the brainstem, we found no benefit at all of EPO in the corpus callosum, with significantly fewer EpoR positive cells present compared to the brainstem. This finding is consistent with the significantly heightened axonal pathology observed in the brainstem, indicating that EPO may be most effective in areas of robust tissue damage, with the highest expression of EpoR previously reported in brain infarct or lesion [80]. Additionally, in this study, we did not see an increase in EpoR numbers in hypoxia only rats in either region examined. Sanchez and colleagues [81] found a 1 hour hypoxic insult of 8% O_2_ was insufficient to stimulate upregulation of EpoR, but a significant effect was achieved when treatment was repeated 3 times over an extended period, indicating that while hypoxia in combination with TAI was sufficient to stimulate EpoR expression, isolated hypoxia may not be adequate.

We then employed immunofluorescence double labelling to determine the cell types staining positively for EpoR. After TAI, expression of EpoR was present predominantly on neurons, with occasional co-localisation with macrophages/microglia. EpoR did not co-localise with astrocytes, or damaged axons. While EPO expression is restricted to neurons and astrocytes only, EpoR is expressed on the cell membrane of neurons, astrocytes, microglia, oligodendrocytes, and endothelial cells [82]. Previous reports have documented heightened expression of astrocytic EpoR under hypoxic conditions both *in vitro* and *in vivo*[65,78,83], and thus, we hypothesised increased numbers of astrocytes expressing the EpoR after TAI + Hx, and even more so in TAI + Hx + EPO rats, as EPO is thought to act in a paracrine manner in the brain [83]; however, this was not corroborated. Contrary to a previous study [84] EpoR staining did not co-localise with neurofilament staining. It is, however, possible that any co-localisation may have been missed using NF-200, as it shows only the most overt axonal damage, whereas the use of light-chain NF may have detected a larger number of axons. Because EpoR expression was observed predominantly in the injured white matter and not on astrocytes or axons, it is possible that EpoR is of an oligodendrocyte or endothelial, or vascular cell origin.

Previously we have demonstrated an early elevation of the cytokines IL-6, IL-1β, and TNF and macrophages/microglia after TAI + Hx [10,15]. This coincided with an increase in inflammatory cell numbers over time as axonal damage subsides, in an effort to clear cellular debris and promote repair [85,86]. However, macrophages and activated microglia also perpetuate secondary damage through the secretion of cytokines, free radicals, and neurotoxic factors such as glutamate [87-89]. Here, early upregulation of IL-1β in the white matter of TAI + Hx rats indicates that IL-1β is produced directly in the most injured tissues. IL-1β promotes macrophage extravasation into damaged tissues [90], as well as the production of other cytokines, prolonging the inflammatory response [91]. Importantly, EPO administration 1 hour after injury was sufficient to reduce IL-1β to sham levels, highlighting EPO’s ability to modulate neuroinflammation early and robustly after diffuse TBI, as reported in models of focal brain injury [26-29]. Although treatment with EPO was unable to reduce IL-1β levels after TAI, there was notably less IL-1β production in these rats. Following early reduction in IL-1β levels, TAI + Hx + EPO rats had a concomitant decrease in the number of infiltrating macrophages and microglia in the corpus callosum at 7 days, and to a lesser extent at 14 days. Interestingly, the same finding did not apply in EPO-treated TAI rats; these rats had a significant increase in macrophages compared to TAI alone at 7 days, and a switch at 14 days to a predominance of microglia. The high number of macrophages at 7 days in TAI + EPO rats is out of proportion to the extent of axonal damage, and particularly so when compared to TAI + Hx rats, and it is possible that this inappropriate cellular immune response at 7 days may be potentiating cerebral inflammation, as evidenced by the high number of microglia at 14 days. This finding has implications for reparative mechanisms and neurological recovery, and certainly requires further investigation to ascertain the mechanisms by which EPO promotes inflammatory cell infiltration/activation after TAI, but not TAI + Hx.

## Conclusions

In this study, we have demonstrated that administration of EPO to rats subjected to the combination of TAI and hypoxia markedly improved behavioural and cognitive performance, attenuated white matter damage, striking resolution of neuronal damage spanning from the axon to the dendrite, and suppressed the neuroinflammatory response, with these results coinciding with enhanced expression of EPO’s cognate receptor EpoR. Fascinatingly, many of these changes occurred after a single injection of EPO, providing compelling evidence of EPO’s ability as a neuroprotective agent. Interestingly, few benefits were observed when EPO was administered to TAI rats without hypoxia, indicating that EPO’s neuroprotective capacity is bolstered under hypoxic conditions, which may be an important consideration when EPO is employed for neuroprotection in the clinic.

## Abbreviations

BBB: Blood brain barrier; Ca2+: Calcium; CCI: Cortical impact injury; CEPO: Carbamylated erythropoietin; CNS: Central nervous system; DAI: Diffuse axonal injury; EPO: Erythropoietin; EpoR: Erythropoietin receptor; HIF-1α: Hypoxia-inducible factor 1 alpha; Hx: Hypoxia; IL-1β: Interleukin-1 beta; i.p.: Intraperitoneally; mmHg: Millimeters of mercury; MRI: Magnetic resonance imaging; NORT: Novel object recognition test; RPM: Revolutions per minute; sham: Sham-operated animal; sO2: Oxygen saturation; TAI: Traumatic axonal injury; TAI + EPO: Traumatic axonal injury with erythropoietin treatment; TAI + Hx: Traumatic axonal injury with hypoxia; TAI + Hx + EPO: Traumatic axonal injury with hypoxia and erythropoietin treatment; TBI: Traumatic brain injury.

## Competing interests

The authors declare that they have no competing interests.

## Authors’ contributions

SCH designed the study, performed all animal work, behavioural testing and immunohistochemistry, drafted the manuscript, and performed statistical analysis. EBY assisted with animal surgery and behaviour and cognitive testing. DSA assisted with animal surgery and behaviour and cognitive testing. NB provided assistance with manuscript preparation, statistical analysis, and figure composition. CMK conceived of the study and oversaw its design and coordination, and drafted the manuscript. All authors have read and approved the final manuscript.
